# A Hybrid CBiGRUPE Model for Accurate Grinding Wheel Wear Prediction

**DOI:** 10.3390/s25092935

**Published:** 2025-05-06

**Authors:** Sumei Si, Deqiang Mu, Hailiang Tang

**Affiliations:** School of Mechanical and Electrical Engineering, Changchun University of Technology, Changchun 130012, China; sisumei88@gmail.com (S.S.); tanghailiang2022@163.com (H.T.)

**Keywords:** CBiGRUPE model, feature extraction, grinding wheel wear, hyperparameter optimization

## Abstract

In grinding machining, monitoring grinding wheel wear is essential for ensuring process quality wear and reducing production costs. This paper presents a hybrid CBiGRUPE model to predict grinding wheel wear, which integrates the advantages of convolutional neural networks (CNNs), bidirectional gated recurrent unit (BiGRU), and the Performer encoder. Time-domain features are extracted from the spindle motor current signals of a surface grinding machine. The structure and hyperparameters of CBiGRUPE are optimized using Bayesian optimization. Experimental validation of the model demonstrates superior performance, with mean absolute error (*MAE*), root mean square error (*RMSE*), and coefficient of determination (*R*^2^) values of 3.041, 3.927, and 0.920, respectively. Compared to models like CNN, BiGRU, and Transformer, the CBiGRUPE model offers more accurate and stable wear predictions. This paper also discusses the advantages and limitations of various models for estimating grinding wheel wear, emphasizing the effectiveness of the proposed approach. This study establishes a foundation for compensating wheel wear and accurately determining the optimal dressing time.

## 1. Introduction

Grinding wheel wear seriously affects processing quality. Therefore, monitoring grinding wheel wear online is essential to ensuring workpiece processing quality, improving processing efficiency, and maintaining production safety [1,2,3].

Currently, grinding wheel wear detection methods are mainly classified into two types: direct and indirect [4]. The direct approach involves using advanced instruments such as a scanning electron microscope [5,6], white light interferometer [7,8], optical microscope [9,10], and optical profilometer [11,12]. While these methods offer high accuracy and comprehensive information, they are costly, require significant downtime, and are limited by the need for a controlled environment, restricting their applicability in real-time production environments. In contrast, the indirect approach evaluates wheel wear by analyzing the characteristics of signals, such as acoustic emission [13,14,15], grinding force [16,17,18], spindle current or power [19,20], and vibration [21,22,23] during the grinding process. This approach supports real-time monitoring, making it suitable for online measurements and significantly improving processing efficiency. As a result, the indirect approach has gained widespread attention in industrial applications due to its ability to monitor grinding wheel wear intelligently.

While the indirect approach enables online monitoring without disrupting production, it still relies on manual feature extraction and struggles to address complex nonlinear relationships in the data effectively. Machine learning and deep learning algorithms have shown promise in predicting grinding wheel wear [24]. Liao et al. [25] proposed a discrete wavelet decomposition-based method to extract the energy features of each raw acoustic emission signal segment and classify the grinding wheel wear state using an adaptive genetic clustering algorithm. Mahata et al. [20] introduced a time-frequency analysis method based on the Hilbert–Huang Transform combined with a support vector machine to monitor the wear state during cylindrical grinding by analyzing vibration and power signals. Shen [26] classified the grinding wheel wear state by analyzing the AE signals generated during the surface grinding of carbon steel workpieces using a support vector machine (SVM) algorithm with a linear kernel. Zhang et al. [27] extracted features from power, acceleration, and acoustic emission signals generated during cylindrical grinding after preprocessing. Then, four features that correlate with grinding wheel wear were selected. Finally, the grinding wheel wear with various process parameters was predicted utilizing an interval two-type fuzzy basis function network (FBFN). Wan et al. [28] employed the whale optimization algorithm to optimize the variational mode decomposition parameters of the AE, vibration, and force signals. Then, the INFO-SVM model was utilized to identify the grinding wheel wear state during surface grinding.

Deep learning models have more robust learning capabilities and better scalability performance than traditional machine learning methods, allowing them to mine valuable information from data. Chen et al. [22] introduced a high-precision identification of grinding wheel wear states in robust noise environments using a combination of the IENEMD noise reduction method, the Hilbert transform, and the one-dimensional convolutional neural network. Guo et al. [29] obtained many features of grinding force, acceleration, and acoustic emission signals by analyzing them during surface grinding, and a two-stage feature selection approach was introduced to obtain the optimal subset of features. In addition, a long short-term memory (LSTM) model was employed to predict grinding wheel wear. Xu et al. [30] presented an approach utilizing variable modal decomposition to reduce noise in acoustic emission signals produced during cylindrical grinding. A neural network model called AO-CNN-LSTM was subsequently employed to assess the wear state. Si et al. [31] proposed a hybrid model called TransBiGRU, which combines the advantages of the transformer encoder and the BiGRU model by analyzing 18 statistical features of current signals to predict wheel wear.

While deep learning models such as CNN, BiGRU, and Transformer perform impressively in various tasks, they still have some limitations. CNNs excel at extracting local features but struggle to capture long-range dependencies within sequences [32]. RNN-based models, like BiGRU, mitigate the vanishing gradient problem but still face challenges with very long sequences and require significant computational resources [33]. Transformer models are highly effective at capturing global dependencies, yet they suffer from memory bottlenecks and high computational complexity, particularly with long sequences [34]. These limitations underscore the necessity for a hybrid approach that combines the strengths of these models while addressing their weaknesses [31,35].

A hybrid model, CBiGRUPE, is proposed to address these limitations, integrating CNN, BiGRU, and the Performer encoder for grinding wheel wear prediction. By combining the strengths of these models, CBiGRUPE captures both local and global features, making it well suited for real-time industrial applications. Compared to other models, such as CNN, BiGRU, and Transformer, the CBiGRUPE model demonstrates superior prediction accuracy and processing efficiency, as shown by lower *MAE* and *RMSE* and higher *R*^2^ values in experimental validation. This paper also discusses the benefits and drawbacks of various models, highlighting the effectiveness of the CBiGRUPE approach in predicting grinding wheel wear.

## 2. Theoretical Foundation

### 2.1. Convolutional Neural Network

**Figure 1:**
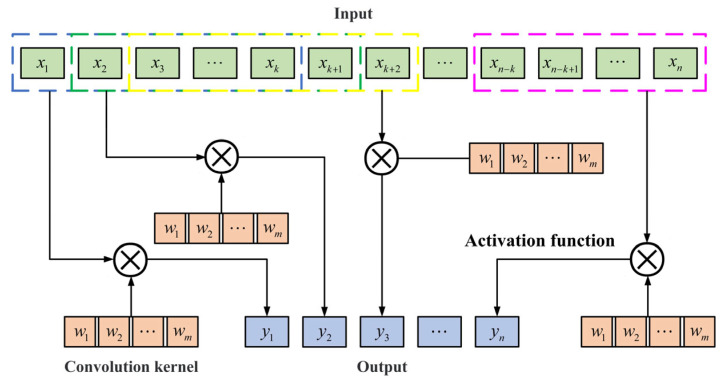
CNN structure diagram.

Grinding wheel wear is a complex process, and a Convolutional Neural Network (CNN) can better extract the information and features related to wheel wear prediction. By moving the convolutional kernel across several input sequence locations, the CNN can effectively capture the sequence data’s varying local patterns and features. As illustrated in Figure 1, the convolutional layer captures local features from the time series data by using a sliding window.

### 2.2. Bidirectional Gated Recurrent Unit

**Figure 2:**
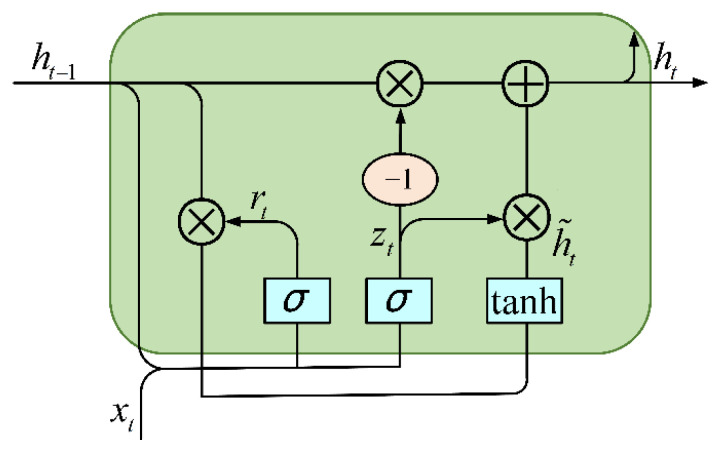
The basic structure of a GRU model.2.3. Performer.

Although the Recurrent Neural Network (RNN) has the advantage of being able to extract contextually relevant information, it can access a minimal range of contextual information and suffers from the problem of long-term dependency. Therefore, it is essential to introduce a threshold mechanism into the RNN structure to preserve the necessary information. It can not only be an effective solution to the issues of gradient vanishing and gradient explosion but also can partially address the shortcomings of the difficulty of transmitting information over long distances. The more commonly used RNN structures with gate mechanisms are Long Short-Term Memory (LSTM) and Gated Recurrent Unit (GRU) architectures, which have temporal solid processing capability. However, because GRU does not need to retain memory, the network’s internal structure has only two gates, which saves memory space, reduces the training parameters, and results in faster training speed. Figure 2 illustrates the basic structure of a GRU model, and the forward GRU computational formula is as follows:

(1) $$r_{t}=\sigma (W_{r}[h_{t-1},x_{t}]+b_{r})$$

(2) $$z_{t}=\sigma (W_{z}[h_{t-1},x_{t}]+b_{z})$$

(3) $${\tilde{h}}_{t}=\tanh(W_{h}[r_{t}*h_{t-1},x_{t}]+b_{h})$$

(4) $$h_{t}=(1-z_{t})*h_{t-1}+z_{t}*{\tilde{h}}_{t})$$

(5) $$y_{t}=soft\max(W_{0}\cdot h_{t}+b_{0})$$

where $x_{t}$ represents the input vector of the current time step; $W_{r}$, $W_{z}$, and $W_{h}$ represent the weight matrices; and $b_{r}$, $b_{z}$, and $b_{h}$ denote the bias vectors.

The BiGRU model can learn both past and the future information. It can learn the input sequence information more comprehensively to avoid missing information when processing long sequences. $g_{t}$ and ${g^{\prime }}_{t}$ denote the corresponding outputs for the Forward GRU layer and Backward GRU layer at moment t, respectively, displayed as follows:

(6) $$g_{t}^{forward}=GRU(x_{t},h_{t-1}^{forward})$$

(7) $$g_{t}^{backward}=GRU(x_{t},h_{t+1}^{backward})$$

The output $O_{t}$ of BiGRU is displayed as follows:

(8) $$O_{t}=\vec{W}g_{t}+\overset{\leftarrow }{W}{g^{\prime }}_{t}+b_{t}$$

where $\vec{W}$ and $\overset{\leftarrow }{W}$ denote the weight matrices for the forward and backward GRU structures, respectively, while $b_{t}$ denotes the bias vector of the output layer.

Performer [36] introduces a linear generalized attention mechanism, which achieves near-linear growth in both space and time complexity by employing the Fast Attention Via Positive Orthogonal Random Features (FAVOR+) algorithm. This approach significantly reduces the training time, particularly when handling long sequences.

The FAVOR+ algorithm defines the attention matrix $A\in {\mathbb{R}}^{L\times L}$ as follows:

(9) $$A(i,j)=K(q_{i}^{T},k_{j}^{T})$$

where $q_{i}$ and $k_{j}$ represents the i−th and j−th row vectors of the query *Q* and key *K*, respectively. The kernel *K* is introduced as follows:

(10) $$K(x,y)=E[\phi {\left(x\right)}^{T}\phi (y)]$$

where ϕ(x) denotes a mapping function.

When $Q^{\prime },K^{\prime }\in {\mathbb{R}}^{L\times p}$, their row vectors are represented as $\phi (q_{i}^{T})$ and $\phi (k_{j}^{T})$, respectively. 

The effective attention, based on the kernel definition, can be expressed as follows:

(11) $${\hat{Att}}_{↔}(Q,K,V)={\hat{D}}^{-1}(BV)$$

where $B=Q^{\prime }{\left(K^{\prime }\right)}^{T}$ and $\hat{D}=diag(B1_{L})$. Here, $\hat{Att}$ denotes the approximate attention, while the brackets indicate the computational order.

**Figure 3:**
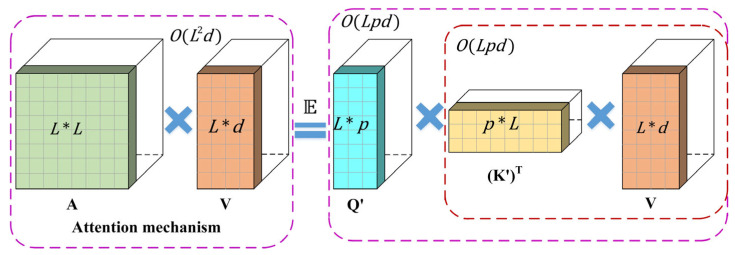
The illustration shows the mechanism of approximate regular attention.

In terms of complexity, the FAVOR+ mechanism achieves a space complexity of O(Lp+Ld+pd) and a time complexity of O(Lpd). In comparison, the conventional attention mechanism has a space complexity of $O(L^{2}+Ld)$ and a time complexity of $O(L^{2}d)$. Figure 3 illustrates the approximate regular attention mechanism.

The mapping function *Φ* of the kernel technique can be defined as follows:

(12) $$\phi (x)=\frac{h(x)}{\sqrt{m}}(f_{1}(\omega_{1}^{T}x),\cdots ,f_{1}(\omega_{m}^{T}x),\cdots ,f_{k}(\omega_{1}^{T}x),\cdots ,f_{k}(\omega_{m}^{T}x))$$

where $f_{1},f_{2},\cdots ,f_{m}$ are different mapping functions, and the random samples $\omega_{1},\omega_{2},\cdots ,\omega_{m}$ are drawn from a distribution *D*. The distribution *D* is isotropic, typically Gaussian.

The softmax kernel of the attention matrix *A* is defined as follows:

(13) $$SM(x,y)=\exp(x^{T}y)$$

If we let $h(x)=\exp({\left\|x\right\|}^{2}/2),l=2,f_{1}=\sin,f_{2}=\cos$, the equation below can be obtained:

(14) $$SM(x,y)=\exp({\left\|x\right\|}^{2}/2)K_{gauss}(x,y)\exp({\left\|y\right\|}^{2}/2)$$

Due to the introduction of sin and cos functions in Equation (13), negative values may arise during the computation. If these negative values emerge after the model has been trained for a particular duration, the resulting variance may become more pronounced, adversely affecting the model’s training process. As a result, the weights needed should ensure that they are all positive to reduce the estimated contrast. The unbiased softmax approximation is achieved by employing the positive random features (PRFs) approach. It ensures that all results are positive, as depicted in Equation (14).

(15) $$\mathrm{SM}(x,y)=E_{\omega \sim N(0,I_{d})}\left[\exp(\omega^{T}x-{\left\|x\right\|}^{2}/2)\exp(\omega^{T}y-{\left\|y\right\|}^{2}/2)E_{\omega \sim N(0,I_{d})}\cosh(\omega^{T}z)\right]$$

where, $N(0,I_{d})$ represents a Gaussian distribution, and cosh denotes the hyperbolic cosine function.

## 3. Proposed Method

**Figure 4:**
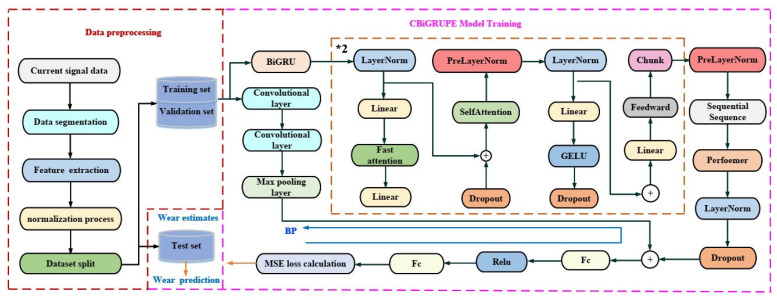
The architecture of the CBiGRUPE model.

Figure 4 illustrates the structure of the CBiGRUPE model, which involves three main parts: data processing, model training, and wear prediction. The *2 symbol indicates that the model contains two identical modules, both enclosed in yellow boxes, which are the same in structure and function.

Data processing: The data processing stage includes noise reduction, feature extraction, and normalization. The initial data are filtered using a moving average filter to identify better patterns associated with wheel wear. This filtering approach removes periodic signals and white noise from the measurement signal. Subsequently, the measurement data are sliced into 200 segments using the upward rounding slicing method. Ten statistical features of the current signals are calculated simultaneously, and these features are normalized.

CBiGRUPE model training and wear prediction: The CBiGRUPE model mainly comprises a CNN module, a Performer encoder module, a BiGRU, a dropout layer, and two fully connected output layers, Fc. The input data first go through the CNN and BiGRU modules simultaneously. The CNN module, in turn, contains convolutional layers, activation functions, and the Maximum Pooling Layer. The CNN model can extract the local features of the data, which can effectively deal with high-dimensional data. The Performer encoder processes the output data generated by the BiGRU model. The multi-layer BiGRU module has better long-term dependency performance and powerful timing processing capability. The Performer encoder contains multiple attentional mechanisms that capture long-distance dependencies in sequence data, enhancing the ability to capture positional correlations in sequences. Skip connections connect the Performer encoder’s output to the CNN module’s output. These additional connections allow for a continuous flow of information, prevent the loss of essential data, and help the model better adjust to the training data, ultimately improving the model’s performance. Dropout reduces the over-reliance of neural networks on training data, thereby improving the model’s generalization capability and reducing the possibility of overfitting. The leave-one-out cross-validation approach is a widely employed method for model evaluation that enhances model reliability, particularly for datasets with limited samples. Moreover, it mitigates the instability caused by random training-validation splits, ensuring more stable evaluation results. The loss function for the CBiGRUPE model is a mean squared error (*MSE*), allowing for effective quantification of prediction errors. Finally, the trained model is used to predict wheel wear, thus facilitating real-time monitoring.

## 4. Experimental Research

### 4.1. Introduction to the Datasets

**Figure 5:**
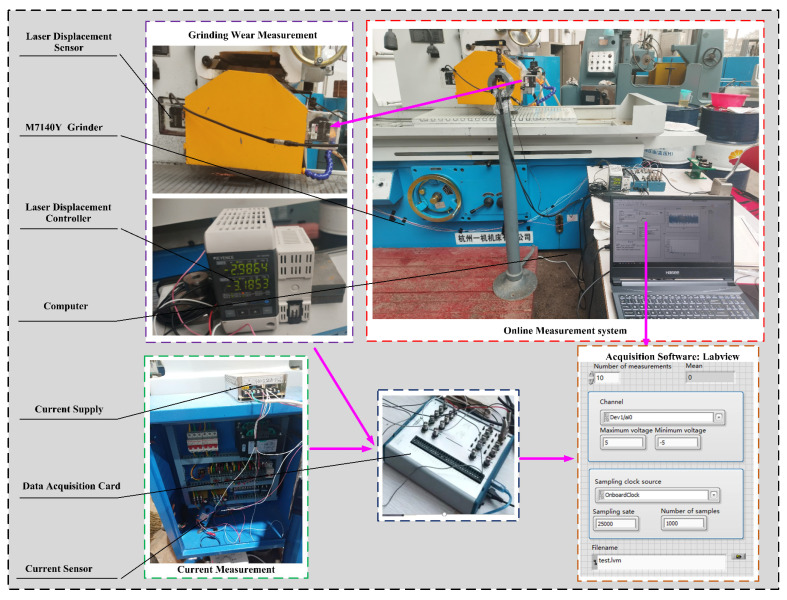
Online measurement system for grinding wheel wear and grinding machine spindle motor current signals.

This study introduces an approach to measure the grinding wheel wear and current signals of the machine tool spindle motor in real time, as depicted in Figure 5. The experiment was finished on surface grinder M7140Y. The selected grinding wheel is a white corundum wheel with a grain size of 46#, which meets the definition of medium grain size according to ISO 6106:2013 [37], balancing material removal rate and surface quality. This choice is consistent with the recommendations for the coarse grinding of carbon steel in the Machinery’s Handbook [38]. Its outer diameter is 350 mm, its inner diameter is 127 mm, and the width is 40 mm, a configuration that effectively balances the need for accurate wear measurement with the operational requirements of the grinding process. The workpiece material used is unquenched No. 45 steel, known for its good machinability and moderate mechanical strength, making it a popular choice for manufacturing mechanical parts. The dimensions of the workpiece are 100 × 18 × 35 mm. The laser displacement sensor records the grinding wheel wear within a linear range of 10 mm and a resolution of 5 μm. This ensures accurate detection of even minor wear changes, suitable for high-precision grinding monitoring. A current sensor records the current signals during grinding. Its input range is 0–10 A AC, and the output range is 0–3 V DC, capable of handling current fluctuations during grinding and providing reliable signals for wear analysis. A 16-bit data acquisition card with a maximum sampling frequency of 500 kS/s was selected to fulfill the experiment’s need for high-frequency data acquisition. NI LabVIEW 2019 was used for data collection. Given the fast fluctuations in signals during the grinding process, the high sampling frequency ensures precise capture of fine details, ensuring data accuracy and timeliness, particularly for dynamic wear prediction.

Because of the unevenness, the grinding wheel must be statically balanced two times before installation to minimize imbalance and reduce vibration that could affect the accuracy of the wear measurements. According to the DIN 12413 [39] grinding wheel safety standards, the grinding wheel speed is 1500 r/min, which falls within the safe operating range for the wheel’s diameter (typically 20–35 m/s). This speed ensures both grinding efficiency and heat management, preventing excessive heat accumulation due to excessively high speeds. The depth of cut is 0.015 mm, providing effective material removal while minimizing excessive wheel wear. This is within the recommended range for coarse grinding in ISO 16089 [40]. The table speed is 50 mm/s, which, in conjunction with the wheel speed and feed rate, ensures smooth and consistent grinding conditions. By appropriately selecting the grinding wheel speed, grinding feed rate, table feed speed, and sensor parameters, the changes in wear can be effectively captured.

After the system setup is completed, since the actual output of the laser displacement sensor may have nonlinear errors, it is necessary to carry out online linear calibration of the laser displacement sensor to ensure that the output of the sensor in the measurement range is linear with the actual distance before carrying out the online detection of the grinding wheel wear amount, to improve the detection accuracy.

**Figure 6:**
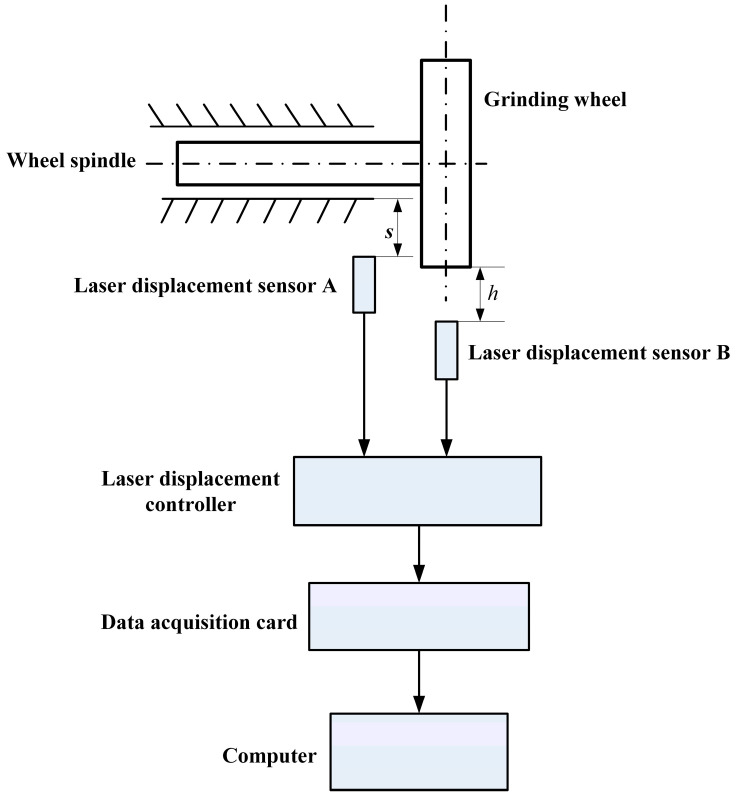
Calibration principle of the online detection system.

The measurement principle of the online detection system is shown in Figure 6. As can be seen from the figure, the position of sensor A is facing a fixed point on the grinding wheel spindle, and its distance to the grinding wheel spindle is s. Sensor B is installed in the position passing through the centerline of the grinding wheel, and its distance to the centerline of the grinding wheel is h. During the grinding process, with the continuous abrasion of the grinding wheel, the gap between the grinding wheel and the laser sensor increases. Therefore, in the calibration experiment, the grinding wheel wear can be simulated by the upward movement of the grinding wheel bracket. The calibration process is as follows:

(1) As shown in Figure 6, adjust sensor A and sensor B so that the distance *s* and *h* are both equal to fixed constant values.

(2) Start the grinding wheel. The number of sampling points for one cycle of grinding wheel rotation is *N*. When the grinding wheel rotates stably, the data are collected by sensor B, and the average value $v_{0}$ of the *N* sampling points is calculated and recorded.

(3) Stop the grinding wheel and manually adjust the grinding wheel bracket upward to increase the reading value of sensor A by Δs, which indicates the amount of wear. At this time, the distance between the grinding wheel and sensor A is $s_{1}$.

(4) Restart the grinding wheel. When the grinding wheel rotates stably, sensor B collects data following the method of step (2), and the average value $v_{1}$ of *N* sampling points is calculated and recorded.

(5) Repeat steps (3) and (4), and when the distance between the grinding wheel and sensor A is $s_{i}$, the corresponding measured value $v_{i}$ is obtained, and the experiment is continued until the end.

(6) The online calibration process detects grinding wheel wear by analyzing and calculating the sampled data.

To eliminate the effect of simple harmonic vibration caused by the unevenness of the grinding wheel, the product of the number of sampling points and the sampling interval time should be a multiple of the whole cycle of the grinding wheel rotation. The speed of the grinding wheel is n=1500 r/min, measured by a tachymeter. The period *T* of the grinding wheel is as follows:

$$T=\frac{1}{n/60}=0.04\;s$$

**Figure 7:**
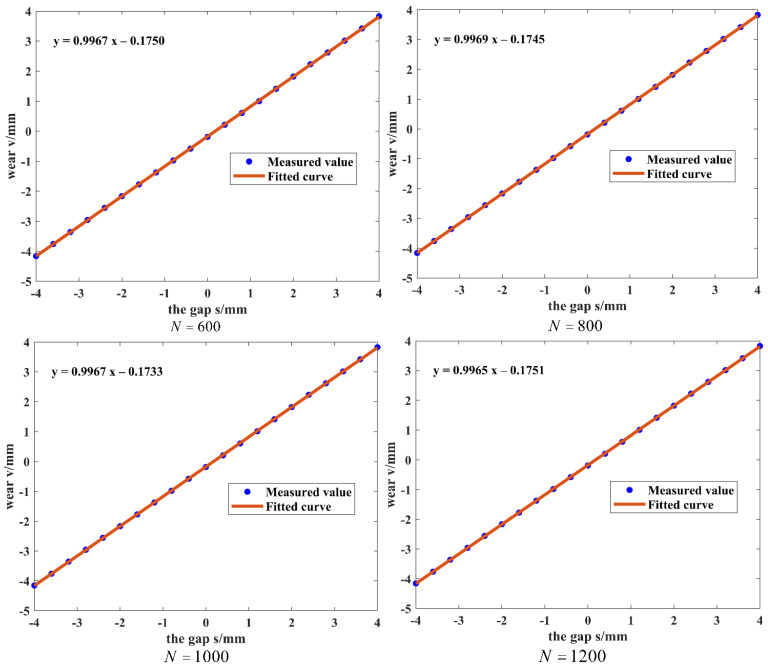
Fitted curves at different sampling points.

The sampling time is set to 10*T* to reduce the influence of random errors on the measurement results. The number of sampling points *N* is selected to be 600, 800, 1000, and 1200 points. The wear amount of each sampling point is represented by the average value of *N* sampling points, and the average value v of the measurement results of 10*T* represents the wear amount of this experiment. The measurement results are shown in Table 1, and the fitting curve is shown in Figure 7.

Based on the measured data in Table 1, the linearity of the system can be calculated when different sampling points are used:

$$\delta_{1}=\frac{\Delta Y_{\max}}{Y}\times 100\%=0.28\%\;(N=600)$$

$$\delta_{2}=\frac{\Delta Y_{\max}}{Y}\times 100\%=0.21\%\;(N=800)$$

$$\delta_{3}=\frac{\Delta Y_{\max}}{Y}\times 100\%=0.20\%\;(N=1000)$$

$$\delta_{4}=\frac{\Delta Y_{\max}}{Y}\times 100\%=0.20\%\;(N=1200)$$

From the above calculations and the fitting equations in Figure 7, it can be seen that the linearity of the fitted curves is 0.28%, 0.21%, 0.20%, and 0.20% and the sensitivities are 0.9967 V/mm, 0.9969 V/mm, 0.9967 V/mm, and 0.9964 V/mm when the number of sampling points is 600, 800, 1000, and 1200, respectively. All are close to 1. This indicates that the system has a good linear relationship between the output and the input and high sensitivity. Therefore, when detecting the wear amount of the grinding wheel, choosing 1000 sampling points can ensure that the system has high linearity and high sensitivity but also has good stability and can obtain a relatively accurate wear amount.

After the linear calibration was completed, to effectively suppress the measurement error caused by mechanical vibration, a special fixture with sealing all around was designed to rigidly fix the laser displacement sensor on the left side of the grinding wheel cover body, away from the coolant nozzle. Further sealing was carried out at the possible gaps in the sensor fixture to prevent the influence of water vapor generated by the high-speed rotation of the grinding wheel on the measurement results. At the same time, to prevent the adverse effects of debris, dust, and coolant splashing generated during the grinding process on the detection accuracy of the laser displacement sensor, a slot was designed between the fixture and the grinding wheel housing, in which a well-elasticized baffle plate was inserted.

When detecting the grinding wheel wear amount online, the number of sampling points is set to 1000, and the sampling time is 10*T*. The specific operation flow is as follows: insert the stopper after feeding and idle for 10 s after the grinding process is completed. Then, remove the stopper and use the LabVIEW program to carry out online detection of grinding wheel wear. Finally, the stopper is inserted again for grinding processing, and the above operation is repeated to realize the online detection of the grinding wheel wear amount.

The experimental steps after the installation of the grinding wheel are as follows:

(1) Turn on the machine and use a single-point diamond tool to dress the grinding wheel. The total amount of dressing is 0.06 mm.

(2) Each feed is followed by reciprocating grinding three times to complete the grinding process. During each grinding stroke, when the grinding wheel touches the workpiece, the current signal is collected for the first two seconds.

(3) Before each subsequent feed, the change in grinding wheel wear is captured by a laser displacement sensor after the completion of each grinding stroke.

(4) Measure and draw the wear curve of the grinding wheel during grinding; according to the drawn curve, the wear can be more easily understood.

(5) Repeat steps (2), (3), and (4) until the grinding wheel is determined to be dull due to a significant change in wear. Then, gather the current signal and grinding wheel wear data to ensure comprehensive dataset coverage. Current signals and the actual wear can be obtained from the dressing to dulling process.

(6) Repeat steps (1), (2), (3), (4), and (5) to obtain three independent repetitions of the experimental datasets N1, N2, and N3. This approach ensures the reproducibility and reliability of the results, providing more robust data for model predictions.

**Figure 8:**
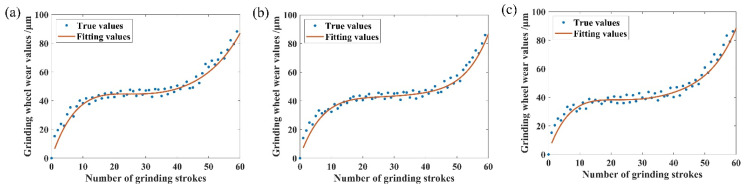
Wear fitting curves: (**a**) fitted curve for N1; (**b**) fitted curve for N2; (**c**) fitted curve for N3.

**Figure 9:**
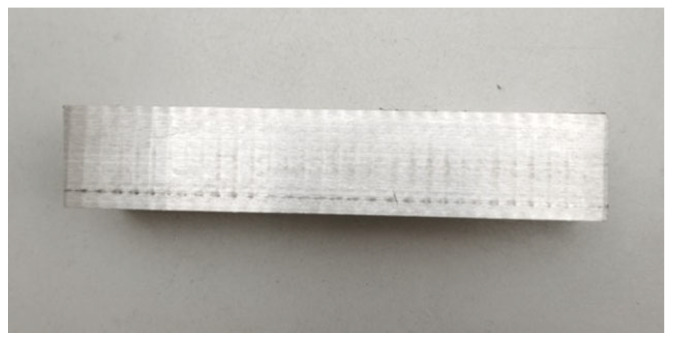
The surface condition of the workpiece at the end of grinding.

Wear curves are derived by fitting a fifth-order polynomial during grinding, as illustrated in Figure 8. This polynomial fitting provides a smooth representation of the wear progression over time. In Figure 8, the sharp abrasive grains on the surface come into contact with the surface of the workpiece for the first time in 0–11 grinding strokes, resulting in more abrasive grains falling off, a high wear rate, and the grinding wheel being in the early stage of wear. During 11–45 grinding strokes, the grinding wheel enters into a relatively stable working state, the wear rate is relatively stable and low, and the grinding wheel is in the normal wear stage. During 45–60 grinding strokes, the abrasive grains of the grinding wheel gradually become blunted, resulting in a significant decrease in cutting ability, indicating that the grinding wheel has entered the normal wear stage. Blunting and reduced cutting ability result in a significantly higher wear rate, causing the grinding wheel to fail and gradually enter the sharp wear stage. A worn wheel may cause vibration marks and damage to the surface of the workpiece due to continuous friction and the heat generated by the friction, as shown in Figure 9.

### 4.2. Feature Extraction

**Figure 10:**
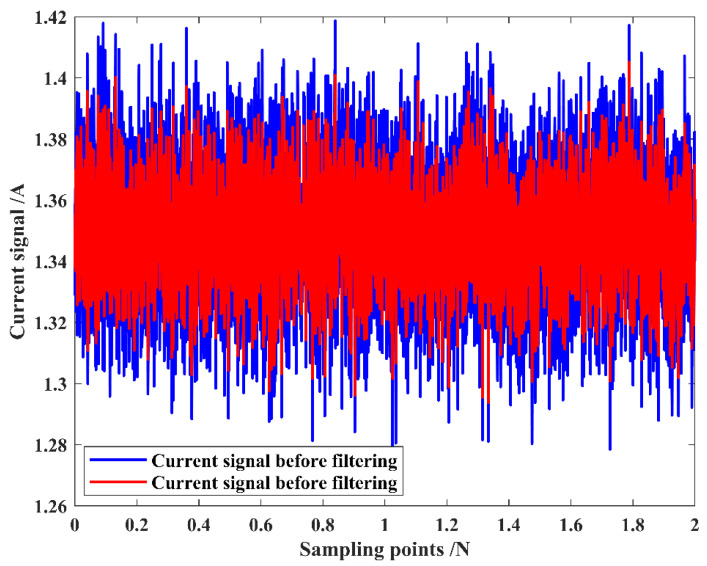
Comparison of current change before and after filtering.

Python 3.11 was used for various stages of data processing, including signal filtering, feature extraction, modeling, and wear prediction. The moving average filtering method filters the collected current signal with a window size k=15, which can better filter out the periodic interference and noise contained in the current signal. The window length of 15 for moving average filtering is chosen for the following reasons: (a) Random noise in the grinding signal usually has a short-term correlation, so choosing an appropriate window length can effectively smooth the noise. Specifically, the window length needs to be larger than the correlation time of the noise to ensure that the filter can effectively remove the high-frequency components of the noise. Setting the window time as too small may result in high-frequency noise not being filtered out, while setting the window time as too large may smooth out the practical feature information in the signal. Therefore, ensuring that the window length covers the correlation time of the noise while avoiding excessive smoothing is a key consideration when choosing the window length. (b) The larger the window length of the moving average filter, the larger the delay, which may affect the real-time performance. Therefore, a window length of 15 is chosen to effectively smooth the noise without introducing too much delay and maintain the signal’s effective characteristics. (c) To further validate the choice of window length, experiments with different window lengths were conducted to compare the signal-to-noise ratio with the correlation time of the noise signal. The results are shown in Table 2. From the table, it can be seen that with the increase in the window length, the signal-to-noise ratio gradually decreases while the correlation time gradually increases. According to the experimental data, when the window length is 15, the signal-to-noise ratio reaches the maximum value of 45.11 dB. Considering the signal-to-noise ratio and the delay together, we choose to obtain the optimal noise-filtering effect and signal retention ability. Selecting the window length of 15 for moving average filtering to filter the acquired current signal can better filter out the periodic interference and noise contained in the current signal. As displayed in Figure 10, the moving average filtering effectively handles the redundant information in the current signal and makes the data smoother.

After signal preprocessing, feature extraction is performed on the current signals. Initially, the segmented current data are divided into 200 non-overlapping segments using an upward rounding slicing method. Ten time-domain features are computed for each segment, as displayed in Table 3. Calculating these time-domain features provides a comprehensive understanding of the distribution, degree of dispersion, concentration trends, and presence of outliers within the data. The extracted features are then normalized.

### 4.3. Optimization of the CBiGRUPE Model

Adam was selected as the optimizer for the proposed model. The Mean Squared Error (*MSE*) is commonly used to evaluate the model’s fitting performance, with a smaller *MSE* indicating better model fitting. Hence, *MSE* is typically utilized as a loss function in a training process and is calculated as follows:

(16) $$MSE=\frac{1}{n}\sum_{i=1}^{n}{\left(y_{i}-{\hat{y}}_{i}\right)}^{2}$$

where *n* represents the sample size, $y_{i}$ denotes the actual values, and ${\hat{y}}_{i}$ indicates the predicted values generated by the model.

**Figure 11:**
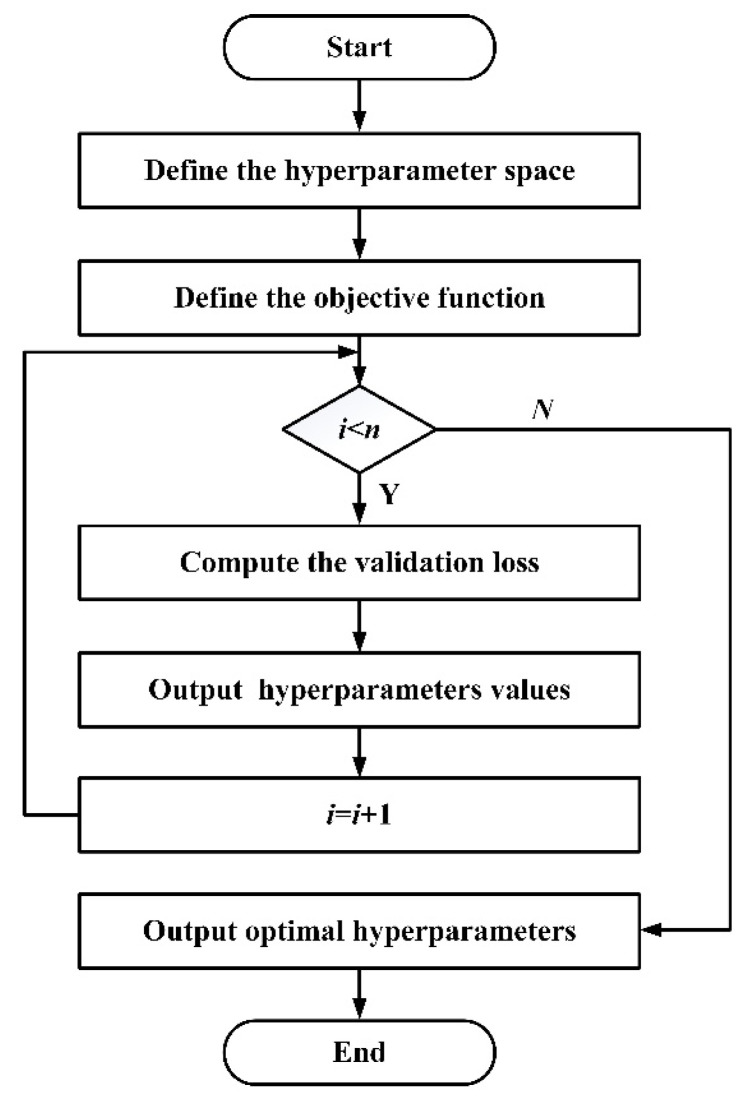
Flowchart of CBiGRUPE model optimization.

Hyperparameter optimization is a critical step in training machine learning and deep learning models, aiming to find the optimal combination of hyperparameters to improve the model’s prediction accuracy. The objective function of the optimization process is the minimum of the average value of the loss function on the validation set:

(17) $$\min f(\theta )=\frac{1}{k}\sum_{i=1}^{k}MSE(\theta ,D_{i})$$

where $\theta =(x_{1},x_{2},x_{3},x_{4},x_{5},x_{6},x_{7},x_{8})$ represents the structural parameters and hyperparameters of the PMSCNN model, *k* denotes the number of samples, and $D_{i}$ represents the validation set data. The parameter search space for Bayesian optimization is shown in Table 4. The structure and hyperparameters of CBiGRUPE are optimized using Bayesian optimization, as presented in Figure 11.

A total of 50 trials were conducted, with an early stopping mechanism implemented to prevent overfitting and save time and computational resources. Table 5 provides a detailed structure of the CBiGRUPE model, while Table 6 lists the optimal hyperparameters.

### 4.4. Evaluation Metrics

*MAE* and *RMSE* are utilized as metrics to assess the performance of predictive models. Additionally, *R*^2^ provides a critical metric for comprehensively evaluating the model’s goodness of fit, providing insights into the proportion of variance the model explains.

(18) $$MAE=\frac{1}{n}\sum_{i=1}^{n}\left|y_{i}-{\hat{y}}_{i}\right|$$

(19) $$RMSE=\sqrt{\frac{1}{n}\sum_{i=1}^{n}{\left(y_{i}-{\hat{y}}_{i}\right)}^{2}}$$

(20) $$R^{2}=1-\frac{\sum_{i=1}^{n}{\left(y_{i}-{\hat{y}}_{i}\right)}^{2}}{\sum_{i=1}^{n}{\left(y_{i}-{\bar{y}}_{i}\right)}^{2}}$$

where *n* represents the sample size, $y_{i}$ denotes the actual values, and ${\hat{y}}_{i}$ indicates the predicted values generated by the model.

### 4.5. Discussion

The leave-one-out cross-validation method [41] can fully use the limited data resources, can improve the model’s generalization ability and assessment accuracy, and is particularly suitable for predicting grinding wheel wear. This study adopted a model integration approach [35] to enhance prediction performance. Integrating the advantages of different models can reduce the bias and overfitting problems in a single model, improving the overall model performance and generalization ability. The structure and hyperparameters of CBiGRUPE are optimized using Bayesian optimization within a specified search space, yielding the optimal hyperparameters.

**Comparison of prediction results based on empirical and optimized parameters:**

A Bayesian optimization approach was used to tune the structure and hyperparameters of the CBiGRUPE model within a specified search space. Table 7 and Table 8 present the evaluation metrics for the prediction results using empirical and optimization parameters on each dataset. As shown in Table 7 and Table 8, the model with optimized parameters outperforms the one with empirical parameters in both *MAE* and *RMSE* metrics. Specifically, in dataset N1, the *MAE* of the optimized model decreases by 0.078 (from 2.590 to 2.512), and the *RMSE* decreases by 0.0101 (from 3.361 to 3.351). Similarly, the N2 and N3 datasets show a similar trend, with the optimized model demonstrating better performance in terms of error reduction. This improvement in prediction accuracy indicates that hyperparameter optimization effectively reduces prediction error and enhances the model’s practical application accuracy.

Regarding model fit, the optimized model generally yields better *R*^2^ values. On the N1 dataset, the *R*^2^ of the optimized model increases slightly from 0.940 to 0.942. However, the most notable improvement is observed in the N2 dataset, where *R*^2^ increases from 0.841 to 0.889. This indicates that the optimized hyperparameters enhance the model’s explanatory power, enabling it to better capture intricate relationships within the datasets.

Overall, the average metrics across all datasets were improved after optimization: *MAE* decreased by 0.251 (from 3.292 to 3.041), *RMSE* decreased by 0.516 (from 4.443 to 3.927), and *R*^2^ increased by 0.025 (from 0.895 to 0.920).

In conclusion, the CBiGRUPE model with optimized parameters achieves a higher prediction accuracy using empirical parameters in all performance indicators. Hyperparameter optimization reduces prediction error, improves model fit, and enhances generalization. It is crucial for enhancing model performance during machine learning development. Future research should explore advanced optimization strategies to improve prediction model efficiency and accuracy.

**Figure 12:**
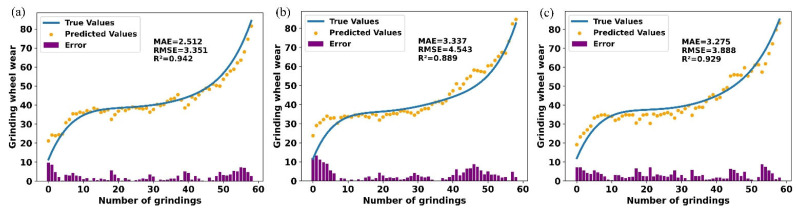
Prediction results for different test sets: (**a**) prediction results for N1; (**b**) prediction results for N2; (**c**) prediction results for N3.

Figure 12 shows the prediction results of the CBiGRUPE model after hyperparameter optimization on various datasets, further verifying the model’s reliable fit and robustness. As shown in Figure 12, the prediction errors remain within a small range, indicating excellent prediction accuracy and confirming the model’s robustness across different datasets.

**Comparison of predictions from different models:**

Analyzing the predictive performance of different models provides insights into their strengths and weaknesses in predicting grinding wheel wear. By leveraging feature selection, different models were optimized for both structure and hyperparameters. Their performance in predicting wheel wear was assessed using the optimized hyperparameters. Table 9, Table 10 and Table 11 demonstrate the evaluation metrics for the prediction results of different models on different datasets.

Statistical significance analysis: The CBiGRUPE model significantly outperforms the CNN model on *MAE* and *RMSE* (p<0.5) and surpasses the CNN and Performer encoder models in *R*^2^ metrics (p<0.5), as confirmed by an independent sample *t*-test (significance level α=0.05). The comprehensive comparison results show that the CBiGRUPE model exhibits superior prediction accuracy in the wheel wear prediction task. Especially in the R^2^ metrics, CBiGRUPE demonstrates significant advantages over the CNN and Performer encoder. While the differences in *MAE* and *RMSE* are not always significant, they are still improved. The stability and accuracy of the overall performance make it highly useful in wear prediction tasks. Therefore, the CBiGRUPE model has a wide range of application prospects in practical industrial applications, especially in wear monitoring and real-time prediction, which can provide accurate and efficient solutions.

CNN model: The CNN model has strong local feature extraction capability, but it is deficient in capturing global dependencies, especially on long time series data, which leads to its low prediction accuracy. This is manifested by MAE=4.455, RMSE=5.547, and $R^{2}=0.834$. On the N2 dataset, CNN’s prediction performance is poor, which may be related to its limited local feature extraction capability and insufficient modeling of global dependencies.

BiGRU model: The BiGRU model has the advantage of capturing forward and backward dependencies but lacks global modeling capability, which makes its performance degraded when dealing with long time series, as shown by MAE=3.547, RMSE=4.502, and $R^{2}=0.890$. However, the BiGRU model exhibits limited global modeling capability, struggling to capture long-range dependencies or global context. Additionally, its sequential nature restricts parallel processing, reducing computational efficiency in large-scale applications.

Transformer model: The transformer model, with its global modeling capability, can capture dependencies in long time series and exhibits high prediction accuracy (MAE=3.103, RMSE=4.171, and $R^{2}=0.907$). However, Transformer has a high computational complexity, the training process requires a large amount of computational resources, the training speed is slow, and the training cost is high in industrial real-time applications. Therefore, in practical applications, Transformer may face the dual challenges of performance and computational resources, especially for the demand of real-time prediction.

Performer encoder model: The Performer model can efficiently capture global dependencies in long-sequence tasks, which can be beneficial for wear prediction. However, its limited focus on local features might affect performance in tasks where local details are crucial (MAE=3.292, RMSE=4.443, and $R^{2}=0.786$).

CBiGRUPE model: The CBiGRUPE model combines the advantages of CNN, BiGRU, and Performer encoder to capture local features and deal with global dependencies in a long time series. The *MAE* (3.041), *RMSE* (3.927), and *R*^2^ (0.920) on all datasets demonstrate superior performance to the other models; especially on the N2 and N3 datasets, the CBiGRUPE model significantly reduces the prediction error and exhibits excellent stability and accuracy. Through this fusion, CBiGRUPE can provide higher accuracy in complex wear prediction tasks, showing significant potential for practical industrial applications and providing a more efficient and accurate solution for wear monitoring in grinding processes.

## 5. Conclusions

This paper proposes an advanced method for predicting grinding wheel wear by analyzing spindle motor current signals, utilizing an optimized CBiGRUPE model. The contributions are listed below:

(1) A laser displacement sensor is employed to measure grinding wheel wear in real time. It provides an efficient and cost-effective solution for online wear monitoring, serving as an alternative to traditional methods.

(2) The CBiGRUPE model, which integrates CNN, BiGRU, and Performer encoders, significantly enhances prediction accuracy by leveraging the strengths of each model, thus improving the overall prediction performance for grinding wheel wear.

(3) The structure and hyperparameters of CBiGRUPE are optimized using Bayesian optimization. This optimization enables the automatic identification of the optimal parameter set, improving the model’s robustness and performance.

(4) The optimized CBiGRUPE model was experimentally validated using spindle motor current signals, achieving impressive average evaluation metrics: MAE=3.041, RMSE=3.927, and $R^{2}=0.920$. These results demonstrate the model’s superior prediction accuracy in predicting grinding wheel wear. A detailed comparative analysis of various models highlights their respective advantages and limitations, offering valuable insights for future optimization.

While this study lays the groundwork for determining wheel dressing time and wheel wear compensation, further research is needed. Future work could integrate force, acceleration, and acoustic emission (AE) sensors to build a more comprehensive dataset, thereby significantly enhancing prediction accuracy. Additionally, the robustness of the CBiGRUPE model under varying grinding parameters and environmental noise should be investigated to further validate its applicability in real-world industrial settings.

## Figures and Tables

**Table 1:** Wear measured at different number of sampling points.

*v*	Sampling Points *N*			
	600	800	1000	1200
4.0	3.82795	3.82719	3.82811	3.82642
3.6	3.42133	3.41895	3.42265	3.41825
3.2	3.01595	3.02081	3.01882	3.01493
2.8	2.61999	2.62202	2.61860	2.62043
2.4	2.23121	2.22907	2.23213	2.22442
2.0	1.81734	1.81760	1.81991	1.81913
1.6	1.41378	1.41111	1.41580	1.41443
1.2	0.99897	1.00930	1.00965	1.00719
0.8	0.60761	0.61414	0.60904	0.61018
0.4	0.21219	0.20790	0.20988	0.20994
0.0	−0.18768	−0.18434	−0.18160	−0.18732
−0.4	−0.57972	−0.57761	−0.57519	−0.57879
−0.8	−0.97386	−0.97705	−0.97669	−0.97847
−1.2	−1.37521	−1.37184	−1.37149	−1.37199
−1.6	−1.77147	−1.77236	−1.77167	−1.77125
−2.0	−2.16405	−2.16527	−2.16647	−2.16538
−2.4	−2.55588	−2.55918	−2.55475	−2.55701
−2.8	−2.95583	−2.95762	−2.95591	−2.95883
−3.2	−3.35892	−3.35758	−3.35489	−3.35798
−3.6	−3.75949	−3.75993	−3.75898	−3.75973
−4.0	−4.15946	−4.15992	−4.15545	−4.15478

**Table 2:** Signal-to-noise ratio and correlation time at different window lengths.

Indicators	15	20	25	30
Signal-to-noise ratio (dB)	46.73	45.11	43.69	42.35
Correlation time (s)	0.00008	0.00012	0.00020	0.00024

**Table 3:** Feature extraction.

Feature	Expression	Feature	Expression
Max	$p_{1}=\max\left\{y_{i}\right\}$	Min	$p_{2}=\min\left\{y_{i}\right\}$
Mean	$p_{3}=\frac{1}{N}\sum_{i=1}^{N}y_{i}$	RMS	$p_{4}=\sqrt{\frac{1}{N}\sum_{i=1}^{N}y_{i}^{2}}$
Standard	$p_{5}=\sqrt{\sum_{i=1}^{N}{\left(y_{i}-p4\right)}^{2}/N}$	Skewness	$p_{6}=\sum_{i=1}^{N}{\left(y_{i}-p4\right)}^{3}/N{\left(p5\right)}^{3}$
Kurtosis	$p_{7}=\sum_{i=1}^{N}{\left(y_{i}-p4\right)}^{4}/N{\left(p5\right)}^{4}$	Peak_to_peak	$p_{8}=p_{1}-p_{2}$
Peaking factors	$p_{9}=p_{1}/p_{4}$	Energy	$p_{10}=\sum_{i=1}^{N}y_{i}^{2}$

**Table 4:** The parameter search space for Bayesian optimization.

Parameter	Search Range
Kernel size of the first convolutional layer	$x_{1}=2,3,4,\cdots ,15$
Number of output channels in the first convolutional layer	$x_{2}=4k,k=1,2,3,\cdots ,32$
Kernel size of the second convolutional layer	$x_{3}=2,3,4,\cdots ,15$
Number of output channels in the second convolutional layer	$x_{4}=4k,k=1,2,3,\cdots ,32$
Kernel size of the maximum pooling layer	$x_{5}=2,3,4,\cdots ,15$
Hidden size of the BiGRU layer	$x_{6}=4k,k=1,2,3,\cdots ,32$
Sizes of the BiGRU layers	$x_{7}=2,3,4$
the dropout rate	$x_{8}\in [0.0,0.5]$
the learning rate	$x_{9}\in [0.00001,0.01]$; it is uniformly distributed in logarithmic space

**Table 5:** Relevant parameters for the CBiGRUPE model.

Layers	Parameters
CNN	Conv1d: in_channels: 10, out_channels: 96, kernel_size: 14, Conv1d: in_channels: 96, out_channels: 44, kernel_size: 8, Activation: ReLU, MaxPool1d: kernel_size:11
BiGRU	input_size: 10, hidden_size: 12, num_layers: 2
Performer encoder	Dimension: 24, Depth: 2, Heads: 4, Attention Head Dimension: 24, Activation: GELU
Fc1	in_features = 14,280, Output features: 64
Fc2	in_features = 64, Output features: 1

**Table 6:** Hyperparameters of the CBiGRUPE model.

Hyperparameters	Batch_Size	Lr	Epoch_Number	Dropout	Max_Patience
values	32	0.00011	200	0.16718	10

**Table 7:** Evaluation metrics of the CBiGRUPE model with empirical parameters.

	Metrics	N1	N2	N3	Mean
Datasets					
*MAE*		2.590	3.905	3.382	3.292
*RMSE*		3.362	5.415	4.553	4.443
*R* ^2^		0.940	0.842	0.902	0.895

**Table 8:** Evaluation metrics of the CBiGRUPE model with optimized parameters.

	Metrics	N1	N2	N3	Mean
Datasets					
*MAE*		2.512	3.337	3.275	3.041
*RMSE*		3.351	4.543	3.888	3.927
*R* ^2^		0.942	0.889	0.929	0.920

**Table 9:** Evaluation metrics for the *MAE* of the different models.

	Datasets	N1	N2	N3	Mean
Models					
CNN		3.909	5.580	3.877	4.455
BiGRU		2.595	4.566	3.480	3.547
Transformer		2.549	3.468	3.292	3.103
Performer encoder		2.590	3.905	3.382	3.292
CBiGRUPE model		**2.512**	**3.337**	**3.275**	**3.041**

**Table 10:** Evaluation metrics for the *RMSE* of the different models.

	Datasets	N1	N2	N3	Mean
Models					
CNN		4.607	7.215	4.818	5.547
BiGRU		3.361	5.851	4.295	4.502
Transformer		3.563	4.690	4.260	4.171
Performer encoder		3.365	5.405	4.548	4.443
CBiGRUPE model		**3.351**	**4.543**	**3.888**	**3.927**

**Table 11:** Evaluation metrics for the *R*^2^ of the different models.

	Datasets	N1	N2	N3	Mean
Models					
CNN		0.891	0.720	0.891	0.834
BiGRU		0.939	0.817	0.913	0.890
Transformer		0.931	0.875	0.915	0.907
Performer encoder		0.840	0.727	0.792	0.786
CBiGRUPE model		**0.942**	**0.889**	**0.929**	**0.920**

## Data Availability

The datasets used during the current study are available from the corresponding author.
